# Perceived neighborhood safety and incident mobility disability among elders: the hazards of poverty

**DOI:** 10.1186/1471-2458-9-162

**Published:** 2009-05-28

**Authors:** Cheryl R Clark, Ichiro Kawachi, Louise Ryan, Karen Ertel, Martha E Fay, Lisa F Berkman

**Affiliations:** 1 Center for Community Health and Health Equity, Division of General Medicine and Primary Care, Brigham and Women's Faulkner Hospitalist Program, Brigham and Women's Hospital, Boston, Massachusetts, USA; 2 Department of Society, Human Development and Health, Harvard School of Public Health, Boston, Massachusetts, USA; 3 Department of Biostatistics, Harvard School of Public Health, Boston, Massachusetts, USA

## Abstract

**Background:**

We investigated whether lack of perceived neighborhood safety due to crime, or living in high crime neighborhoods was associated with incident mobility disability in elderly populations. We hypothesized that low-income elders and elders at retirement age (65 – 74) would be at greatest risk of mobility disability onset in the face of perceived or measured crime-related safety hazards.

**Methods:**

We conducted the study in the New Haven Established Populations for Epidemiologic Studies of the Elderly (EPESE), a longitudinal cohort study of community-dwelling elders aged 65 and older who were residents of New Haven, Connecticut in 1982. Elders were interviewed beginning in 1982 to assess mobility (ability to climb stairs and walk a half mile), perceptions of their neighborhood safety due to crime, annual household income, lifestyle characteristics (smoking, alcohol use, physical activity), and the presence of chronic co-morbid conditions. Additionally, we collected baseline data on neighborhood crime events from the New Haven Register newspaper in 1982 to measure local area crime rates at the census tract level.

**Results:**

At baseline in 1982, 1,884 elders were without mobility disability. After 8 years of follow-up, perceiving safety hazards was associated with increased risk of mobility disability among elders at retirement age whose incomes were below the federal poverty line (HR 1.56, 95% CI 1.02 – 2.37). No effect of perceived safety hazards was found among elders at retirement age whose incomes were above the poverty line. No effect of living in neighborhoods with high crime rates (measured by newspaper reports) was found in any sub-group.

**Conclusion:**

Perceiving a safety hazard due to neighborhood crime was associated with increased risk of incident mobility disability among impoverished elders near retirement age. Consistent with prior literature, retirement age appears to be a vulnerable period with respect to the effect of neighborhood conditions on elder health. Community violence prevention activities should address perceived safety among vulnerable populations, such as low-income elders at retirement age, to reduce future risks of mobility disability.

## Background

Preventing the onset of mobility disability among elders is a public health priority in the United States (US) [1]. Generally, disability can be defined as difficulty or dependency in performing roles and tasks needed for independent living and self-care [2]. Mobility disability, an early sign of the disablement process, is defined as difficulty or dependency in functioning due to decreased walking ability, maneuverability, or speed [3,4]. Mobility disability often predicts the onset of more severe functional impairment, such as Activities of Daily Living (ADL) disability [5]. Though the incidence of disability in the US is decreasing, the absolute number of disabled older adults is projected to increase as the population ages [1,6]. Growing numbers of aging-related disability episodes are expected to increase public costs of care and reduce quality of life for those affected [2]. Thus, identifying population-based factors that trigger the disablement process is important to promoting healthy aging.

To this end, the growing socioeconomic status (SES) disparity in aging-related disability among elders is of particular concern [7,8]. Schoeni et al. report widening SES disparities in the prevalence of disability among older adults in the National Health Interview Survey [8]. Between 1982 and 2002, only small declines in the annual prevalence of disability were seen among low-income elders (-1.38%) compared to higher-income groups (-3.1%). Moreover, using data from the 2000 Census Supplementary Survey, Minkler et al. show a persistent SES gradient in the risk for mobility limitations, with highest risks among elders with low incomes at 150% of the poverty level and below [9]. The connections between low SES and mobility disability are not fully understood. In the US, the associations between low income, for example, and poor health outcomes are attributed to *psychosocial conditions *(e.g., low position in social hierarchy, high levels of stress, fewer opportunities for social engagement), *lifestyle behaviors *(e.g., smoking, heavy alcohol use), and *material resources *(e.g., poor access to health insurance, poor-quality housing, poor-quality neighborhoods) [7,10-12]. Among lower-income elders, these factors are thought to promote the development of chronic co-morbid conditions and present environmental challenges that trigger and advance the disablement process [3,13]. Finding specific factors that contribute to risks among low-SES elders is an active area of inquiry.

It is possible that chronic exposure to dangerous neighborhoods may have implications for the onset of mobility disability among low-income elders. Living in dangerous or high-crime neighborhoods is frequently cited as a potential health hazard for low-income elders [7]. An emerging literature examines aspects of disadvantaged neighborhoods (perceived safety, crime, walkability, SES of neighbors) that may promote the onset of mobility disability among low-SES elders [14]. Summary scores and indices that measure aspects of disadvantaged neighborhoods have been associated with risks for mobility disability onset among middle-aged and older adults in cross-sectional and longitudinal studies [15-18]. However, these studies have not found specific associations between measures of perceived neighborhood safety or neighborhood crime rates and the onset of mobility disability. To our knowledge, direct measures of neighborhood crime rates and individuals' perceptions of safety from crime have not been investigated together in longitudinal studies of mobility disability onset among low-income elders.

Theoretically, chronic exposure to neighborhood crime may contribute to stress, allostatic load, and the onset of co-morbidity. Second, though elders are less frequently victimized by crime than younger adults, crime may expose elders to risk of direct injury leading to mobility disability onset [19]. In addition, lack of perceived neighborhood safety could constrain health-promoting behaviors such as walking, or increase negative coping behaviors such as smoking or alcohol use [20,21]. Moreover, neighborhoods with high crime rates or a reputation for being "dangerous" may have more difficulty attracting businesses that provide material resources and services. A longitudinal study of how crime rates versus perception of safety affect low-income elders may give insights into whether dangerous neighborhoods "get into the body" to initiate the disablement process and how this might occur (perception of safety versus measured crime level). We note that prior studies of neighborhood safety investigated the impact of safety over short time periods, among relatively high-income cohorts, and focused on either young or broad age groups. National survey data indicate that neighborhood conditions have their greatest effect on adults near retirement age, and may be weak or non-existent among middle-aged adults, and the oldest old [9,15,22].

Thus, here we examine effects of neighborhood crime rates and perceived neighborhood safety hazards due to crime in a longitudinal cohort of retirement-aged and older elders free from mobility disability in the New Haven Established Populations for Epidemiologic Studies of the Elderly (EPESE). We hypothesize that over an eight-year period, elders who live in high-crime neighborhoods and those who perceive their neighborhoods as unsafe due to crime at baseline will have higher risk of an incident mobility disability event than those who do not. Additionally, we hypothesize that these risks will be particularly salient in low-income populations who are at risk for high exposure and have fewer resources for coping with stress.

## Methods

### Sample

The New Haven EPESE is a longitudinal cohort of 2,812 non-institutionalized elders aged 65 and older in 1982. New Haven is an urban city in Connecticut with a socio-economically diverse population. The EPESE study design and sampling frame have been described elsewhere [23]. Briefly, the New Haven EPESE used a stratified probability sampling scheme to survey elders in three non-institutionalized housing settings: in public (government-subsidized) housing, age-segregated elder community (non-subsidized) housing, and non-age segregated community housing [23]. Men and African Americans were over-sampled to achieve adequate numbers. The New Haven EPESE achieved a response rate of 82%.

Data were collected annually; face-to-face interviews were conducted at baseline and at three-year intervals, and telephone surveys were conducted in the intervening years. Participants in the present investigation were followed for eight years from baseline in 1982 or until death. Data on deaths were obtained through matching participants with the National Center for Health Statistics National Death Index.

### Measures

#### Mobility disability

Mobility disability, an early marker of elder disablement, was defined as the inability to climb a flight of stairs or walk a half-mile without assistance, based on the work of Guralnik et al [3]. Mobility disability was assessed through two survey questions that defined "assistance" as the need for special equipment or help from other people. Elders who reported losing the ability to perform either task (walking up and down stairs, or walking a half-mile without assistance) at an annual follow-up interview were coded as having a mobility disability in the year they reported that functional loss.

#### Perceived neighborhood safety

Perceived neighborhood safety at baseline was measured with the item "how safe from crime would you say your neighborhood is?" This item was also used in the Behavioral Risk Factor Surveillance Survey (BRFSS) [20]. Participants were considered to have perceived a safety hazard if they felt "not too safe," or "not safe at all," and were considered unexposed if they felt "very safe," "fairly safe," or "somewhat safe"[20]. Categorizing "somewhat safe" as unexposed provides a conservative measure of exposure to perceived safety hazards.

#### Ecologic data: neighborhood crime rates

Crime statistics at the neighborhood level were not readily available from public sources in New Haven for the baseline time period in 1982. Thus, the authors collected crime reports meeting Uniform Crime Reports (UCR) category definitions from the city's major newspaper, the New Haven Register (NHR), to derive neighborhood crime rates. Though there is no gold standard for assessing crime rates at the neighborhood level, the definitions of UCR crime categories (homicides, aggravated assaults, rapes, robberies, burglaries, larcenies, motor vehicle thefts, and arsons) are used consistently across government reporting agencies and are considered a valid and reliable index of the types of crime residents view as serious events [24]. To obtain our measure of neighborhood crime, we defined neighborhoods as census tracts. The boundaries of the 28 census tracts (neighborhoods) in New Haven were obtained from the 1980 US Census. We abstracted the addresses of UCR crimes reported in the NHR at baseline in 1982 and geocoded the addresses of UCR crime events to the census tract level with ArcView GIS 3.2^®^. The number of UCR crimes per square mile of the census tract reported in the NHR was used to measure neighborhood crime rates (NHR Crimes). Using the PROC RANK procedure in SAS^®^, neighborhoods were then ranked by the number of NHR Crimes per square mile in the neighborhood (census tract) and divided into "highest-crime neighborhoods" (highest tertile) and lower-crime "comparison neighborhoods" (first and second tertiles). To determine whether our measure of neighborhood crime was at all related to impressions of exposure to crime among neighborhood residents, we calculated the percentage of EPESE participants who perceived neighborhood safety hazards due to crime at baseline within each census tract, and then calculated the ecologic correlation between the NHR Crime rate measure in each census tract, and the percentage of EPESE participants who perceived neighborhood safety hazards due to crime in each census tract.

#### Socioeconomic status: income

The participant's baseline annual household income from self-reported wages, salaries, social security, retirement benefits, help from relatives, and rent from property was coded into low ($0 – $4,999), middle ($5,000 – $9,999), and high ($10,000 and greater) income groups. The federal poverty line in 1980 was approximately $5,000 among those aged 65 and older for a household of two {2007 108/id}. We defined low-SES (impoverished) elders as those below the poverty line. We compared impoverished elders ($0 – $4,999) to middle and highest-income groups ($5,000 and greater).

#### Residential tenure and moving from baseline neighborhoods

An indicator variable denoted the number of years elders lived in their baseline neighborhoods (≤ 5 years or >5 years). A separate variable indicated whether they moved away from the baseline residence at any point during the study period.

#### Demographics

Race/ethnicity, sex, and age were assessed by self-report. This analysis was restricted to participants who identified as non-Hispanic black or non-Hispanic white, and excludes 43 New Haven EPESE participants from other or unidentified race/ethnic groups. Age categories were divided into retirement age (ages 65 to 74) and older elders (ages 75 and older).

#### Co-morbidities

We measured self-reported *co-morbid conditions *at baseline as confounders of the relationship between neighborhood safety (perceptions of safety and crime rates) and mobility disability. Elders were asked to report whether a physician had given a diagnosis of a specific chronic medical condition. A co-morbidity index was then created as the sum of self-reported conditions present at baseline [3,26]. Conditions correlated with incident mobility disability in prior literature were included in the index (myocardial infarction, stroke, hypertension, diabetes mellitus, arthritis, hip fracture, cirrhosis, and cancer). Myocardial infarction (MI) episodes are thought to have a strong correlation to mobility disability, and multiple MI events were treated as separate events and increased the co-morbidity score [3]. Obese body mass index (BMI) was included as a separate variable; a BMI greater than or equal to 30 was used to define obesity.

Additionally, we used validated scales to measure *cognitive impairment *and *depressive symptoms *as factors that may influence perceptions of neighborhood safety. The 6-item Short-Portable Mental Status Questionnaire (SPMSQ) measured cognitive function [27]. Inability to answer three or more questions correctly denoted cognitive impairment. The Center for Epidemiologic Studies Depression Scale (CES-D) measured depressive symptoms; scores of 16 or higher were considered to indicate depression [28,29].

#### Physical activity: walking behavior

To assess potential mediating effects of neighborhood safety on physical activity, self-reported walking behavior was assessed. Respondents reported subjective walking habits as "often," "sometimes," or "never" during the month prior to the interview.

#### Smoking and alcohol

To assess potentially negative coping strategies, we measured smoking behavior and alcohol use. Smoking status was measured by self-report of never, ever, or current smoking through two questions: "Do you smoke cigarettes now?" and "Did you ever smoke cigarettes?" Non-smokers were identified as those who responded that they did now, nor ever smoke cigarettes. Thirty-day alcohol consumption was calculated using self-reported daily intake of cans of beer, ale, glasses of wine, and spirits over the past month. Monthly ounces of alcohol were divided into three categories: (1) none (2) moderate (≤ 70 oz. per month), and (3) heavy consumption (> 70 oz. per month). Prior EPESE findings show that moderate alcohol consumption has protective cardiovascular effects [30].

### Statistical Analysis

The analysis excluded participants with mobility disability at baseline, participants with missing data on baseline mobility status, and one participant lost to follow up, for whom the incident mobility disability event could not be identified. Time to incident mobility disability within eight years of follow-up was calculated from annual survey responses between 1982 and 1990. Participants were censored in the year they reported an incident mobility disability event, and stopped contributing person-time to the analysis from that year forward. Elders who died prior to experiencing an incident mobility event were censored as a non-event and stopped contributing person-time to the analysis at the time of death.

Descriptive statistics and bivariate associations for continuous and categorical variables at baseline were estimated with the PROC DESCRIPT and PROC CROSSTAB procedures in SAS-callable SUDAAN^® ^to obtain variances estimated with the Taylor Series approximation with replacement (WR) and were weighted with survey design weights to account for the complex EPESE sampling frame. Age-adjusted incidence rates representing the total number of incident cases of mobility disability over eight-years of follow up per 1,000 person-years were calculated in SUDAAN with the PROC RATIO procedure.

Hazard Ratios (HR) with 95% confidence intervals (CI) were computed with proportional hazards models with discrete time intervals in SUDAAN to estimate effects of perceived neighborhood safety hazards or living in the highest-crime neighborhoods at baseline on incident mobility disability in the full cohort, and among subgroups of elders at retirement age (65 to 74 years), older elders at ages 75 and older, as well as among impoverished elders and higher-income groups [31]. Models were then adjusted for co-morbidities and lifestyle characteristics that may confound or mediate potential effects of perceived neighborhood safety or living in high-crime neighborhoods. Tests for statistical interaction between age and poverty status, and between age and perceived neighborhood safety or crime were also performed. Analyses were conducted in SAS^® ^version 9.2. The Harvard School of Public Health Institutional Review Board approved this study.

## Results

### Cohort characteristics

Baseline cohort characteristics are shown in Table 1, with weighted percentages reported. The majority of the cohort was retirement-age, 65 to 74 years (65%). The cohort was predominantly female (59%), white (84%), and most had annual household incomes above the federal poverty line (73%). At baseline, the cohort was generally healthy; only 35% had more than one co-morbid condition. Sixteen percent of the cohort was obese. Few had a significant cognitive impairment (4%). The prevalence of depression was 12%.

**Table 1:** Baseline characteristics. Percentage of study participants who perceive the neighborhood is unsafe due to crime. (N = 1884)^†^

	Total N(%)	Perceives neighborhood safety hazard due to crime N (%)	SE	P
**Age**				
65–74	1152 (65)	249 (22)	1.62	0.54
75 and older	732 (35)	157 (20)	2.50	
**Gender**				
Male	892 (41)	197 (20)	1.77	0.35
Female	992 (59)	209 (22)	1.88	
**Race**				
Non-Hispanic Black	343 (16)	103 (34)*	4.03	< 0.001
Non-Hispanic White	1541 (84)	303 (19)*	1.49	
**Annual household income**				
Above 1980 federal poverty line	1086 (73)	217 (20)	1.64	0.06
Below poverty line	583 (27)	136 (26)	2.92	
**Co-morbid conditions**				
Zero or one	1207(65)	240 (20)	1.69	0.19
Two or more	677 (35)	166 (24)	2.26	
**Obesity (BMI ≥ 30 kg/m^2^)**				
Obese	247 (16)	49 (19)	3.12	0.49
Not obese	1533 (84)	330 (22)	1.54	
**Cognitive impairment**				
None or mild impairment	1782 (96)	389 (22)	1.47	0.27
Significant impairment	91 (4)	15 (16)	5.28	
**Depression**				
None or mild symptoms	1627 (88)	314 (19)*	1.50	< 0.001
Depressed	220 (12)	81 (36)*	3.98	
**Smoking history**				
Current	381 (18.7)	81 (21)	2.82	0.88
Former	566 (28.8)	132 (22)	2.34	
Never	934 (52.5)	193 (21)	1.86	
**Alcohol use**				
None	843 (43)	183 (22)	2.05	0.88
Moderate	925 (52)	196 (21)	1.91	
Heavy (> 70)	96 (5)	21 (24)	5.43	
**Walks for physical activity**				
Often	1053 (54)	224 (21)	1.82	0.06
Sometimes	536 (29)	108 (18)	2.26	
Never	282 (17)	72 (28)	3.38	
**Number of years lived at baseline address**				
5 years or less	727 (23)	147 (19)	2.19	0.17
Greater than 5 years	1145 (77)	257 (22)	1.66	
**Changed residence**				
Did not move from baseline address	1282 (66)	262 (19)*	1.64	0.02
Moved from baseline address at any point during study	602 (34)	144 (26)*	2.38	
**NHR Crime rate**				
Highest crime neighborhood (top tertile)	506 (27)	175 (33)*	3.52	< 0.001
Crime in comparison neighborhoods (1^st ^– 2^nd ^tertiles)	1378 (73)	231 (17)*	1.28	
**Perceives neighborhood safety hazard due to crime**	406 (22)	-	-	-

^†^Weighted percentages are calculated in SUDAAN. No participants had a mobility disability at baseline. Totals do not sum to 100% where data are missing due to item non-response. Missing data on perception of safety N = 45 (2%); Missing data on income = 215 (11%). Cochran-Mantel-Haenszel test of proportions in SUDAAN accounts for sample weights. *P < 0.05.

With respect to lifestyle, approximately 19% were current smokers, and 5% were heavy alcohol drinkers (more than 70 ounces per month, or more than four drinks daily). The cohort was physically active, with 54% rating the subjective frequency of their walking behavior as "often" in the past month.

The majority of elders were long-time residents in their baseline homes; 77% lived in their baseline homes for more than five years. Thirty-four percent moved from their baseline residences at some point over the eight-year study period. Twenty-seven percent of study participants lived in the highest-crime neighborhoods, and 22% perceived their neighborhoods were unsafe due to crime (Table 1).

### Perceived neighborhood safety hazards and NHR Crime: ecological associations

**Figure 1:**
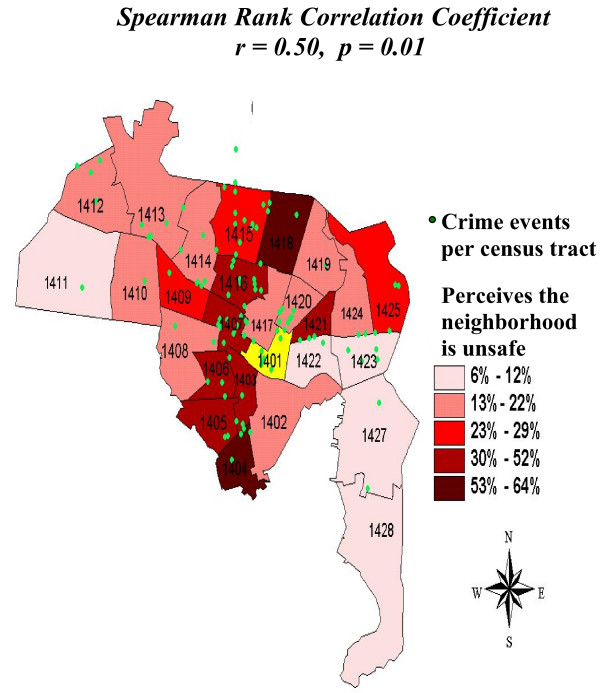
**Percentage of residents who believe the neighborhood is unsafe due to crime**. Displays percentage of New Haven EPESE participants who perceived their neighborhood was unsafe due to crime in 1982. Green overlay represents crime events per census tract in 1982. Neighborhoods are measured as individual census tracts. Census tracts are numbered 1401–1428. Tract 1401 = Central Business District. Source data: (1) New Haven Established Populations for Epidemiologic Studies of the Elderly (EPESE) 1982. (2) New Haven Register crime event reports for year 1982.

Figure 1 shows the geographic distribution of NHR Crime events and the percentage of EPESE residents reporting a perceived safety hazard due to crime in New Haven neighborhoods, defined as census tracts. Seventy-nine percent of NHR Crimes (116 of 147 New Haven neighborhood crimes) reported in the newspaper were successfully geocoded; 21% had too little reported address information to assign the event to a specific census tract. The median number of crimes per square mile was 4.9 crimes, ranging from zero crimes to 37.2 crimes per square mile (inter-quartile range 1.45 – 12.57 crimes per square mile). To assess whether any correlation existed between the NHR Crime rate assessment and observations of local EPESE residents, Spearman rank correlation coefficients were computed among 24 New Haven neighborhoods with stable boundaries between 1980 and 1990, excluding three neighborhoods with few EPESE participants (<10) within the census tract. A statistically significant ecologic correlation was seen between the NHR Crime rate and the percentage of EPESE residents who perceived neighborhood safety hazards due to crime (n = 24, r = 0.50, p = 0.01). The Spearman correlation including all 28 census tracts was also statistically significant (n = 28, r = 0.46, p = 0.01).

### Correlates of perceived neighborhood safety hazards

Table 1 describes the baseline associations between perceived neighborhood safety hazards and covariates measured at study entry among individual study participants. Those who lived in the highest-crime neighborhoods at baseline (assessed by newspaper reports) were more likely to perceive their neighborhoods as unsafe due to crime than those in the comparison neighborhoods (33% vs. 17%, p < 0.001). Black study participants were more likely than whites to perceive their neighborhoods as unsafe (34% vs. 19% p < 0.001). Similarly, those with significant depressive symptoms were more likely to perceive their neighborhoods were unsafe than those with no or mild symptoms (36% vs. 19%, p < 0.001). Those who moved from their baseline residence at any point during the study were more likely to have reported their neighborhoods were unsafe due to crime at baseline than those who did not move (26% vs. 19%, p < 0.05). Impoverished elders tended to report their neighborhoods as unsafe due to crime more frequently than those with incomes above the poverty line (26% vs. 20%), though this was not statistically significant (p = 0.06). Those who reported they never walked for exercise tended to report a perceived neighborhood safety hazard (28%) more frequently than those who rated their walking behavior as "sometimes" (18%) or often (21%), though this association not statistically significant (p = 0.06).

### Correlates of living in the highest-crime neighborhoods

**Figure 2:**
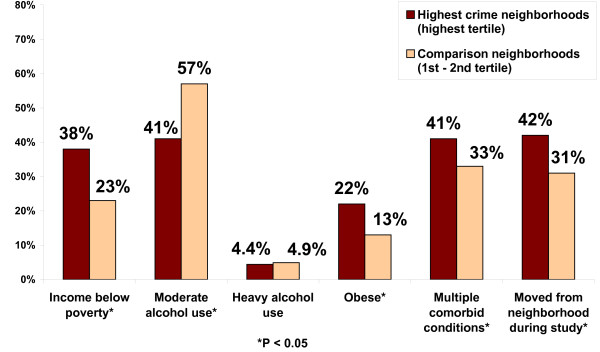
**Correlates of living in the highest-crime neighborhoods**.

Figure 2 shows selected correlates of living in the highest-crime neighborhoods at baseline (neighborhoods ranked in the highest crime tertile by the NHR Crime rate) compared to the "comparison" neighborhoods (ranked in the second and lowest crime tertiles by the NHR Crime rate). The highest-crime neighborhoods had a higher prevalence of impoverished elders than the comparison neighborhoods (38% vs. 23%, p < 0.01). Strikingly, Blacks were much more likely than whites to live in the highest-crime neighborhoods (68% vs. 19%, p <0.0001). Elders in the highest-crime neighborhoods were less likely to engage in moderate alcohol use than those in the comparison neighborhoods (41% vs. 57%, p < 0.0001) but did not appear to have a higher percentage of heavy alcohol use (4.4% vs. 4.9%). Those living in the highest-crime neighborhoods were more likely to be obese (22% vs. 13%, p < 0.05), have multiple co-morbid conditions (41% vs. 33%, p < 0.05), and have significant cognitive impairment (6% vs. 3%, p < 0.05) than those in the comparison neighborhoods. Those in the highest-crime neighborhoods were also more likely to have moved from their baseline neighborhoods during the study period than those in the comparison neighborhoods (42% vs. 31%, p < 0.01).

### Age-adjusted incident mobility disability rates: relation to poverty, perceived safety and neighborhood crime exposure

Table 2 shows the age-adjusted incidence of mobility disability within eight years of follow-up associated with each mobility disability risk factor in the complete cohort, unadjusted for other risk factors. Neither perceived neighborhood safety hazards nor living in the highest crime neighborhoods were associated with mobility disability in the complete cohort. Having an annual income below the federal poverty line was associated with an increased risk of mobility disability in the complete cohort (HR 1.31, 95% CI 1.08 – 1.59). Female sex, multiple co-morbid conditions, obesity, depression, cognitive impairment, current smoking, and moving from the baseline residence during the study period were also statistically significantly associated with an increased risk of mobility disability at the end of the eight-year follow up period in the complete cohort. Having walked often for exercise in the past month, moderate alcohol intake, and long-term tenure (> 5 years) in the baseline residence were associated with reduced risk of mobility disability in the complete cohort.

**Table 2:** Eight-year age-adjusted mobility disability incidence rates per 1,000 person years and corresponding age-adjusted hazard ratios

	**Rate**	**95% CI**	**SE**	**HR (95% CI)**
**Non-Hispanic Black**	148	125 – 172	0.012	1.15 (0.91 – 1.45)
Non-Hispanic White	130	119 – 142	0.006	Reference
**Female**	151	136 – 166	0.008	1.33 (1.13 – 1.55)**
Male	107	95 – 119	0.006	Reference
**Below poverty**	156	133 – 178	0.011	1.31 (1.08 – 1.59)**
Above poverty	120	108 – 131	0.006	Reference
**Perceives neighborhood safety hazard due to crime**	134	113 – 155	0.011	1.02 (0.83 – 1.25)
Does not perceive safety hazard	132	120 – 144	0.006	Reference
**Lives in highest crime neighborhoods**	146	122 – 170	0.012	1.16 (0.95 – 1.42)
Lower crime comparison neighborhoods	128	117 – 140	0.006	Reference
**Multiple Co-morbid Conditions**	171	149 – 193	0.011	1.59 (1.34 – 1.88)**
One or no Co-morbid Conditions	117	106 – 127	0.005	Reference
**Obese**	164	123 – 206	0.021	1.32 (1.02 – 1.70)*
Not obese	127	116 – 138	0.005	Reference
**Depressed**	174	140 – 208	0.017	1.37 (1.08 – 1.73)**
None or mild depressive symptoms	127	116 – 138	0.005	Reference
**Cognitive Impairment**	312	179 – 445	0.068	1.71 (1.15 – 2.54)**
No cognitive Impairment	130	119 – 140	0.005	Reference
**Current smoker**	147	124 – 170	0.012	1.27 (1.03 – 1.57)*
Former smoker	118	100 – 136	0.009	0.96 (0.79 – 1.16)
Never smoked	133	119 – 147	0.007	Reference
**Walked "often" for exercise in past month**	118	106 – 130	0.006	0.78 (0.64 – 0.95)*
"Sometimes" walked for exercise	142	120 – 165	0.012	Reference
"Never" walked for exercise	171	143 – 198	0.014	1.18 (0.92 – 1.52)
**No alcohol use**	151	134 – 168	0.009	Reference
Moderate alcohol intake	121	108 – 133	0.006	0.76 (0.65 – 0.90)**
Heavy alcohol intake	113	73 – 154	0.021	0.83 (0.56 – 1.22)
**Moved from baseline residence during the eight year study period**	168	150 – 187	0.009	1.41 (1.20 – 1.66)**
Did not move during study period	113	101 – 125	0.006	Reference
**More than five years in baseline residence**	125	114 – 137	0.006	0.73 (0.61 – 0.87)**
Five years or less in baseline residence	164	141 – 188	0.012	Reference

Table presents the incident cases of mobility disability per 1,000 person-years among participants at risk for a first mobility disability event between years 1982 and 1990. Rates are age-adjusted within decades (65–69 yrs, 70 – 79 yrs, 80 – 89 yrs, and 90 yrs and older). Standard errors (SE) and 95% confidence intervals (CI) are calculated in SUDAAN (log rates and standard errors are not shown). Age-adjusted univariate hazard ratios estimating risk of incident mobility disability over eight-years associated with each listed variable and reference group (not adjusted for other covariates) are calculated in proportional hazard models with discrete time intervals. *P < 0.05, **P < 0.01

### Predictors of mobility disability: hazards of poverty and lack of perceived safety among retirement-aged elders

Table 3 shows poverty as a predictor of incident mobility disability over eight years of follow-up, in age, race and sex adjusted models. An increased risk of incident mobility disability associated with poverty was seen among elders at retirement age (HR 1.32, 95% CI 1.02 – 1.70), but not among older elders (HR 1.10, 95% CI 0.82 – 1.47). There was no statistically significant multiplicative interaction between age and poverty.

**Table 3:** Eight-year age-specific incident mobility disability rates and hazard ratios for the effect of poverty

	Aged 65–74		Aged 75 and older	
	Rate (SE)	HR (95% CI)	Rate (SE)	HR (95% CI)
Below poverty	120 (0.013)	1.32 (1.02 – 1.70)*	204 (0.024)	1.10 (0.82 – 1.47)
Above poverty	81 (0.006)	Reference	176 (0.013)	Reference

Table presents age-specific rates for incident mobility disability events per 1,000 person-years among those at risk for an incident event between years 1982 and 1990. Hazard ratios and 95% confidence intervals (CI) associated with poverty are adjusted for race, sex, and age as a continuous variable.

*P < 0.05.

There was no statistically significant effect of perceived neighborhood safety on eight-year incident mobility disability in the complete cohort (Table 1). However, a statistically significant multiplicative interaction term was found between the effect of age and perceived safety hazards (HR 1.57, 95% CI 1.06 – 2.34). Stratified models in Table 4 show an increased risk of mobility disability associated with perceived neighborhood safety hazards among retirement-aged elders with incomes below the poverty line (HR 1.69, 95% CI 1.06 – 2.69), but not among retirement-aged elders above the poverty line. A trend toward a *decreased *risk of mobility disability associated with perceived safety hazards was seen among older elders (Table 4). No statistically significant effect of living in the highest-crime neighborhoods was seen in any group.

**Table 4:** Effects of perceived neighborhood safety and living in highest-crime neighborhoods^†‡^

	Aged 65–74 Above poverty line	Aged 65–74 Below poverty line	Aged 75 and older Above poverty line	Aged 75 and older Below poverty line
	Rate (SE)	Rate (SE)	Rate (SE)	Rate (SE)
Perceives neighborhood safety hazard due to crime	87 (0.012)	183 (0.034)	139 (0.027)	151 (0.035)
Does not perceive neighborhood safety hazard due to crime	80 (0.007)	102 (0.013)	188 (0.015)	217 (0.029)
	HR (95% CI)	HR (95% CI)	HR (95% CI)	HR (95% CI)
Effect of perceived neighborhood safety hazard^†^	1.12 (0.81 – 1.55)	1.69 (1.06 – 2.69)*	0.69 (0.45 – 1.05)	0.65 (0.38 – 1.12)
	Rate (SE)	Rate (SE)	Rate (SE)	Rate (SE)

Lives in highest-crime neighborhoods	89 (0.019)	155 (0.031)	176 (0.029)	209 (0.036)
Lives in lower crime comparison neighborhoods	80 (0.006)	105 (0.013)	176 (0.014)	201 (0.031)
	HR (95% CI)	HR (95% CI)	HR (95% CI)	HR (95% CI)
Effect of living in highest crime neighborhoods^‡^	1.18 (0.72 – 1.94)	1.36 (0.85 – 2.17)	1.18 (0.77 – 1.81)	1.09 (0.73 – 1.62)

Table presents eight-year incident mobility disability rates per 1,000 person-years.

^†^Hazard ratio and 95% confidence intervals (CI) for the effect of perceived neighborhood safety hazards, adjusted for age, race and sex.

^‡^Hazard ratio and 95% confidence intervals (CI) for the effect of living in highest crime neighborhoods (highest tertile of NHR crimes) adjusted for age, race and sex.

*P < 0.05.

### Predictors of mobility disability adjusted for covariates

Multivariable models estimating the effects of perceived neighborhood safety hazards were stratified by age and poverty status to determine whether effects of perceived safety hazards could be explained by covariates (Table 5). There was no statistically significant effect of perceived neighborhood safety hazards (HR 1.31 95% CI 0.94 – 1.82) or of living in the highest-crime neighborhoods (HR 1.10, 95% CI 0.70 – 1.71) among retirement-aged elders with incomes above the poverty line. The statistically significant predictors of mobility disability among retirement-aged elders above poverty were having multiple co-morbid conditions, smoking at baseline, having been active at baseline by walking often in the past month, and long-time residential tenure in the baseline residence.

**Table 5:** Hazard ratios associated with incident mobility disability: by age and poverty status.

	Aged 65 to 74 Above poverty line	Age 65 to 74 Below poverty line	Age 75 and older Above poverty line	Age 75 and older Below poverty line
Black race	0.60 (0.33 – 1.09)	1.29 (0.81 – 2.05)	0.62 (0.30 – 1.28)	0.52 (0.24 – 1.11)
Age (continuous variable)	1.12 (1.06 – 1.19)*	1.17 (1.08 – 1.26)*	1.12 (1.07 – 1.18)*	1.13 (1.06 – 1.20)*
Female sex	1.09 (0.80 – 1.50)	1.02 (0.61 – 1.73)	1.34 (0.91 – 1.98)	1.12 (0.59 – 2.14)
Perceives neighborhood safety hazard due to crime	1.31 (0.94 – 1.82)	1.56 (1.02 – 2.37)*	0.55 (0.36 – 0.85)*	0.67 (0.36 – 1.25)
Lives in highest crime neighborhoods	1.10 (0.70 – 1.71)	1.33 (0.80 – 2.21)	1.55 (0.93 – 2.59)	1.03 (0.66 – 1.60)
Multiple Co-morbid Conditions	1.54 (1.15 – 2.07)*	2.05 (1.37 – 3.07)*	1.06 (0.73 – 1.56)	2.19 (1.13 – 4.23)*
Obese	1.44 (0.99 – 2.10)	0.84 (0.47 – 1.48)	1.47 (0.79 – 2.77)	2.96 (1.33 – 6.60)*
Depression	1.59 (0.98 – 2.58)	1.12 (0.57 – 2.17)	0.90 (0.56 – 1.44)	0.76 (0.42 – 1.37)
Cognitive Impairment	1.84 (0.39 – 8.70)	1.92 (0.95 – 3.86)	1.40 (0.30 – 6.58)	2.92 (1.28 – 6.64)*
Current smoker	1.51 (1.03 – 2.23)*	1.69 (0.99 – 2.86)	1.17 (0.69 – 2.00)	1.36 (0.52 – 3.54)
Former smoker	0.92 (0.62 – 1.37)	1.36 (0.82 – 2.27)	0.90 (0.54 – 1.49)	0.73 (0.32 – 1.69)
Walked "often" for exercise in past month	0.59 (0.43 – 0.82)*	0.76 (0.42 – 1.38)	1.01 (0.65 – 1.58)	0.92 (0.48 – 1.76)
"Never" walked for exercise in past month	0.73 (0.45 – 1.19)	1.53 (0.89 – 2.66)	1.81 (1.06 – 3.11)*	0.75 (0.33 – 1.69)
Moderate alcohol intake	0.86 (0.63 – 1.16)	0.88 (0.56 – 1.38)	0.82 (0.57 – 1.17)	0.55 (0.33 – 0.92)*
Heavy alcohol intake	1.30 (0.79 – 2.14)	1.20 (0.36 – 4.08)	0.90 (0.37 – 2.21)	0.65 (0.14 – 2.96)
Moved from baseline residence during the eight year study period	1.29 (0.94 – 1.76)	1.62 (1.01 – 2.60)*	1.45 (1.07 – 1.95)*	1.42 (0.92 – 2.17)
More than five years in baseline residence	0.54 (0.40 – 0.73)*	1.44 (0.99 – 2.08)	0.72 (0.50 – 1.03)	0.64 (0.41 – 0.99)*

Table presents proportional hazards models fully adjusted for listed covariates. Reference groups: Non-Hispanic White; male sex; does not perceive neighborhood safety hazard due to crime; lives in lower crime comparison neighborhoods (1^st ^and 2^nd ^tertiles); zero or one co-morbid condition; not obese (< 30 kg/m^2^); none or mild depressive symptoms; no cognitive impairment; never smoked; "sometimes" walked for exercise in past month; no alcohol intake; did not move from baseline residence during the study period; lived five years of less in baseline residence. *P < 0.05

However, among retirement-aged elders who were impoverished, perceiving a safety hazard was a statistically significant predictor of mobility disability after adjustment for covariates (HR 1.56, 95% 1.02 – 2.37). Living in the highest-crime neighborhoods did not predict incident mobility disability events in this sub-group. Having multiple co-morbid conditions and having moved from the baseline residence during the study predicted an increased risk of incident mobility disability over the eight-years of follow-up among impoverished retirement-aged elders. However, long-time residential tenure in the baseline residence was associated with a non-statistically significant trend toward an *increased *risk of mobility disability in this group (HR 1.44, 95% CI 0.99 – 2.08). Taking walks was not statistically significantly associated with reduced mobility disability risk among impoverished retirement-aged elders.

### Associations among older elders

In the adjusted model among elders aged 75 and older, those with incomes above the poverty line who perceived their neighborhoods were unsafe due to crime had a *decreased *risk of incident mobility disability compared to those who did not perceive safety hazards (HR 0.55 95% CI 0.36 – 0.85). Additionally, elders aged 75 and older with incomes above the poverty line had increased risks of mobility disability if they moved from their baseline residence, or reported never walking for exercise in the past month prior to study entry.

No statistically significant effect of perceived safety hazards was found among impoverished elders aged 75 and older. Correlates of mobility risk among impoverished elders aged 75 and older included having multiple co-morbid conditions, being obese, and having a significant cognitive impairment (Table 5). Long-term residential tenure predicted a lower risk of incident mobility disability among impoverished elders aged 75 and older compared to those with short-term residential tenure (≤ 5 years in the baseline residence). Moderate alcohol use was also protective in this sub-group.

No statistically significant effects of living in the highest-crime neighborhoods were found among elders aged 75 and older, regardless of poverty status.

## Discussion

Our study tested the hypotheses that perceiving neighborhood safety hazards due to crime, or living in high-crime neighborhoods would increase the risk of incident mobility disability, particularly among low-income elders. We found that perceiving safety hazards increased the risk of incident mobility disability among impoverished elders at retirement-age, and that this effect was not completely explained by baseline lifestyle behaviors and co-morbid conditions. In contrast, we found no evidence for an effect of perceived safety hazards among retirement-aged elders with incomes above the poverty line. With respect to neighborhood crime, we found: (1) strong correlations at the ecologic level between our measure of the neighborhood crime rate and the percentage of EPESE residents who believed their neighborhoods were unsafe due to crime, (2) strong associations between an individual's lack of perceived safety and residence in neighborhoods with the highest crime levels, as well as (3) significant cross-sectional associations between an individual's residence in the highest-crime neighborhoods and having multiple co-morbid conditions at baseline. Nonetheless, we found that unlike perceiving safety hazards, living in the highest-crime neighborhoods did not predict incident mobility disability in any group. Finally, we found an inverse association by which older elders above the poverty line appeared to have a *decreased *risk of incident mobility disability when they perceived their neighborhoods were unsafe at baseline, compared to those who did not perceive a safety hazard.

Our findings among low-income elders at retirement age (65 to 74) support observations in the literature that neighborhood conditions may have the greatest negative effects on this age group [22]. Robert and Li evaluated data from the Americans' Changing Lives survey and the Midlife Development in the United States study, and found that relationships between community context and measures of health in aging populations (self-rated health, number of chronic conditions) are strongest at retirement age measured at age 60 to 69 in their analyses. The retirement age period may mark a vulnerable life-stage that is made more stressful by neighborhood contextual factors that breed insecurity, thereby predisposing elders to future mobility limitation. Studies of the peri-retirement period suggest that elders may experience marked changes in social identity, as well as changes in feelings of self-worth and self-esteem that could create psychosocial stress [32,33]. Moreover, the peri-retirement period may also mark a change in access to an individual's financial resources, such as income [32].

Our findings contrast with previous studies that have not found strong effects of perceived neighborhood safety or measured crime rates. Two studies measured perceived neighborhood safety as a separate item in relation to mobility disability incidence. Among these, neither the Alameda County Study (a prospective one-year analysis of elders aged 55 and older) nor the African American Health Study (a three-year study of middle-aged adults aged 49–65) found an association between perceived neighborhood safety and incident mobility disability [17,18]. Direct measures of crime rates were not assessed in these studies, and the impact of perceived safety within low-SES groups was not a specific focus. A recent cross-sectional investigation in the US Health and Retirement Study examined the effect of government-reported Uniform Crime Report (UCR) crime rates as part of a composite measure of disadvantaged neighborhoods (county-level UCR crime rates and Black residential segregation) among elders aged 55 and older. No effect of crime/segregation on prevalent mobility disability was found, and measures of perceived neighborhood safety were not available [15]. The positive findings in our study likely reflect the longitudinal design, the focus on age-specific effects, and the focus on low-income populations who may be most vulnerable to perceptions of neighborhood conditions. Our data support the hypothesis that the negative effect of perceived safety is strongest among retirement-aged elders who are impoverished, and who have fewer resources to buffer the effects of neighborhood conditions compared to higher income elders. Significantly, among impoverished retirement-aged elders, we did not find protective factors that may mitigate against mobility disability. While higher-income elders at retirement age enjoyed the benefits of frequent walking and long-term tenure in their baseline homes, we did not find these factors to be protective among elders who were impoverished at retirement age. In fact, long-term residential tenure tended to increase risk of developing mobility disability among these elders.

The finding that perception of safety, rather than crime rates, was related to incident mobility disability in this group generates the hypothesis that dangerous neighborhoods "get into the body" to engender mobility disability through psychosocial or psychological processes. Effects of perceived safety were robust to adjustment for cognitive impairment and depressive symptoms, both of which may increase risks for mobility disability or influence one's appraisal of neighborhood conditions. Strong correlations between living in the highest-crime neighborhoods and perceiving safety hazards among individual study participants suggest that study participants' perceptions were grounded in observable neighborhood conditions related to crime. But at the same time, perceptions more than objective measures appeared to correlate with future mobility disability. Further studies using survey data or biologic data that capture stress-related pathways that may connect perceptions of safety to disablement processes would help us understand the mechanisms underlying these associations.

With respect to the older elders in this cohort (75 and older), "inverse" associations between perceptions of safety and mobility disability should not be discounted. Previous research by Lachs et al. among EPESE participants identified an inverse cross-sectional relationship between ADL disability and criminal victimization, by which older adults who were victimized by crime were *less *likely to have ADL disabilities than those who were not victimized by crime [19,34]. Lachs et al. posit that elders with ADL disabilities may be less likely to travel about their neighborhoods, and therefore, may be less likely to experience unsafe conditions or be victimized by crime. It is also possible that inverse associations between perceived safety hazards or victimization among the oldest elders may indicate selection bias. This is to say, among the oldest adults, psychosocial effects of unsafe neighborhoods, or victimization from crime, may selectively lead to death as a competing risk, rather than disablement, such that survivors appear relatively healthy. Moreover, one might also reason that the oldest adults who perceive their neighborhoods are unsafe may have developed strategies for stress reduction, or for avoiding hazards, that protect them from disablement processes. Future research in other cohorts should examine effects among the oldest old to determine whether cross-over (inverse) effects can be detected in relation to neighborhood safety hazards, or other measures of neighborhood disadvantage. For example, in the Asset and Health Dynamics among the Oldest Old (AHEAD) study, chronic disease cross-over effects associated with race were seen around age 76, and ADL disability cross-over effects associated with race were seen at age 86 [35]. Further research may identify age cross-over points with respect to neighborhood safety hazards as demonstrated in the EPESE cohort.

Taken together, our findings suggest that the effects of neighborhood safety on mobility disability operate through elders' perceptions rather than through direct measures of crime, and that such negative perceptions of safety pose a hazard primarily for retirement-aged elders who are poor. Competing risks of mortality may complicate interpreting findings among the oldest old.

Our findings should be interpreted in light of limitations in our data. First, in studying perceived safety, we cannot fully eliminate self-report bias as a contributor to our findings, where those who are developing mobility disability perceive their environments to be less safe due to crime. The prospective study design, limiting the cohort to those mobile at baseline, and adjusting for co-morbid conditions makes reverse causation unlikely as a sole explanation for our findings. Second, due to the difficulty in finding crime statistics for small areas, we obtained our measure of neighborhood crime rates from the city's newspaper. Though journalists work with local law enforcement to find source material, media reports of crime events are known to be biased toward severe and dramatic events, and only a small sample of crimes are reported [36,37]. However, the strong ecologic correlation between the NHR Crime rate and the percentage of EPESE residents who felt unsafe due to crime provides some indication that NHR reported crime events may have also been observable by local residents, lending some corroborating evidence that we have classified neighborhoods appropriately. The lack of associations we report may point to the limitations of crime assessed at the census tract level as a measure of neighborhood exposure, i.e., a differential misclassification measurement error, where larger census tracts may be internally heterogeneous, and less accurately described than smaller areas. Future studies with data accurate to smaller areas than census tracts (i.e., specific blocks) may reveal associations that our data are unable to test.

An additional limitation is that we used annual household income to measure of SES, rather than an index of wealth that estimates assets and net worth, which are thought to capture socioeconomic status more completely than income in elderly populations [38]. Moreover, because elders in the EPESE cohort have relatively low incomes, we were not able to identify an SES gradient effect of income on either mobility disability or on susceptibility to a perceived neighborhood safety hazard. Furthermore, we used a crude measure of poverty status that could not adjust for household size. Noting these limitations, we observed a strong threshold effect by which retirement-aged elders in poverty had increased risk of incident mobility disability compared with elders with incomes above poverty. To explain the effect of low income on disability, one might speculate that even elders with some wealth or assets, but low incomes, may continue to face the risk of disability, as elders with low incomes may hesitate to spend proceeds from their assets when replacement income is less available.

An additional limitation, in contrast to the existing literature on the effects of neighborhood disadvantage on health outcomes, we measured two facets of disadvantage, perceived neighborhood safety and neighborhood crime, rather than a larger multifaceted index of disadvantage with other observed conditions such as neighborhood walkability. Indices of neighborhood disadvantage may be considered more holistic appraisals of neighborhood conditions, since they assess multiple dimensions known to affect disablement, including social and built environmental characteristics [39]. To be sure, neighborhoods with high crime or low perceived safety among residents are rarely without other aspects of disadvantage, including low-SES or impoverished neighbors, noise, traffic, and fewer health-promoting resources [18,21,40]. On the other hand, identifying specific exposures connected to mobility disability can help target tailored interventions for at-risk communities. The issue of teasing out confounding to understand the individual contributions of built environmental characteristics and social determinants, which are likely highly collinear in most low-SES contexts, may be best approached through intervention research designed to mitigate the risk of one facet of disadvantage at a time. Our study contributes to this effort by suggesting that intervening in the psychosocial dimension of perceived safety may be useful among elders. However, our study is limited by statistical power (i.e., a small number of neighborhoods) and unable to observe relationships among neighborhood-level factors in an observational or multi-level context.

Last, a potential limitation is that observations of the EPESE cohort were made in the past, from the 1980s to 1990s. Given the small declines in disability prevalence among low-income elders, and persistent SES gradients in risks for disability between this period and the present, the experiences of low-SES elders in this cohort remain quite relevant to understanding contextual factors that contribute to mobility disability risks. We note that during this period, the New Haven EPESE cohort had few participants who were from racial/ethnic backgrounds other than non-Hispanic blacks and whites, and we do not present this data here. Additional cohort studies are needed to determine if our results generalize to other racial and ethnic groups.

These important limitations considered, our study has several strengths, including a longitudinal prospective design, a community-based random sample of elders with varied residential exposures, a high response rate (82%), and the use of previously validated measures of mobility disability and safety perception. Moreover, the analysis detected diversity in effects of socioeconomic factors and facets of neighborhood disadvantage in at least two stages of the life course, with evidence for heightened vulnerability at retirement age. Finally, our findings on the effect of a perceived safety hazard on incident mobility disability risk have implications for future public health interventions. For example, community-based interventions might survey low-SES elders at retirement age, and target social capital building, social support, or other appropriate interventions toward elders who feel unsafe in their neighborhoods as strategies to reduce risk for disablement [41]. Whereas violence prevention programs are frequently targeted toward youth, our study suggests that interventions targeted toward improving safety perception in populations such as low-SES retirement-aged elders may reduce morbidity associated with aging.

## Conclusion

We find that among elders who are impoverished at retirement age, perceiving neighborhood safety hazards increases their risk of incident mobility disability. Additionally, we suggest further investigations of the correlates of perceived safety among the oldest old may help us understand factors that promote healthy aging. Our findings underscore the importance of the psychosocial dimension of neighborhood context as a determinant of healthy aging. Specifically intervening to improve perceptions of neighborhood safety at retirement age may be an important step to reduce the risk of mobility disability among vulnerable elders.

## Competing interests

The authors declare that they have no competing interests.

## Authors' contributions

CRC conceived the analyses, designed and carried out the analyses, prepared the manuscript, and reviewed and approved the final version. IK conceived and contributed to the design of the ecologic data collection, and contributed to the conception of the analysis, and preparation of the manuscript. LR contributed to the design and interpretation of the analysis. KE contributed to conducting the analysis. MEF contributed to the data collection, design and data management of the analysis. LFB contributed to the parent study design and data collection, the conception and design of the present analysis, and preparation of the manuscript. All authors reviewed, edited, and approved the final version of the manuscript.

## Pre-publication history

The pre-publication history for this paper can be accessed here:


